# Risk of African Swine Fever Virus Sylvatic Establishment and Spillover to Domestic Swine in the United States

**DOI:** 10.1089/vbz.2018.2386

**Published:** 2019-06-26

**Authors:** Jillian D. Wormington, Andrew Golnar, Karen C. Poh, Rebekah C. Kading, Estelle Martin, Sarah A. Hamer, Gabriel L. Hamer

**Affiliations:** ^1^ Department of Veterinary Integrative Biosciences, Texas A&M University, College Station, Texas.; ^2^ Department of Entomology, Texas A&M University, College Station, Texas.; ^3^ Department of Microbiology Immunology and Pathology, Colorado State University, Fort Collins, Colorado.

**Keywords:** Argasidae, *Ornithodoros*, co-occurrence, *Sus scrofa*

## Abstract

African swine fever virus (ASFV) causes a high-consequence foreign animal disease that has emerged along international trade routes. Owing to high lethality and resulting trade sanctions, establishment of this disease in the United States would have devastating economic consequences. ASFV can be transmitted by soft ticks in the genus *Ornithodoros* or directly between swine, including domestic, feral, and wild swine. Consequently, the spatial risk of ASFV establishment depends on where susceptible animals, with or without competent vectors, co-occur. We synthesized county-level historical records of soft tick occurrence, current maps of feral swine distribution, and domestic swine inventory to evaluate the risk of ASFV establishment and spillover in the United States. Areas of California, Florida, and much of the southwestern United States were classified as high risk for ASFV establishment and spillover should an introduction event occur. Our analyses indicate that California, Texas, Georgia, and Florida are high-priority candidates for proactive risk reduction strategies. Domestic swine are often produced in high-biosecurity environments, mitigating health risks associated with contacting infected hosts and vectors. However, small-scale and organic pig producers in much of the southern United States remain more vulnerable to disease emergence.

## Introduction

African swine fever (ASF) is a devastating disease in pigs associated with serious economic consequences due to high lethality, culling, and resulting trade sanctions with unaffected countries (Sánchez-Cordón et al. 2018). ASF is caused by the African swine fever virus (ASFV), a member of the Asfarviridae family (*Asfivirus* genus). In domestic swine, ASFV causes high fever, lethargy, digestive dysfunction, respiratory distress, and nasal discharge, resulting in death 7–10 days after symptoms arise (Penrith and Vosloo 2009). Most viral strains cause nearly 100% mortality; subclinically infected or recovered animals may shed virus for over a month, necessitating depopulation of potentially affected populations.

Since ASFV was first described in Kenya in 1921, it has been discovered in 25 other African countries and has sporadically emerged throughout Europe and the Americas (Penrith et al. 2013). In 1957, an epizootic occurred in Portugal, followed by a series of other incursions into European countries, including France, Italy, Belgium, the Netherlands, and Spain (Biront et al. 1987). The virus later spread to Cuba, Brazil, Haiti, and the Dominican Republic in the Western Hemisphere (Butler and Gibbs 1984). ASFV was eradicated from most areas outside of Africa in the mid 1990s, but the virus remains an issue in the Italian island of Sardinia. A second transcontinental spread to Georgia in the Russian Caucasus in 2007 grew to impact Eastern European countries, including Russia, Ukraine, Poland, Latvia, Lithuania, Estonia, Moldova, Czech Republic, and Romania (Sánchez-Cordón et al. 2018), where the virus remains present today.

The ability of ASFV to spread through international trade networks represents a significant risk to the global agricultural industry. The United States supports a large domestic swine industry, with sales in 2012 of $22.5 billion USD (USDA-NASS 2012). Given the costs associated with ASFV outbreaks—equivalent to >$250 in 1999 USD per affected pig (Rendleman and Spinelli 1999)—prevention and early intervention are key to mitigating this invasive threat.

ASFV can be transmitted to feral or domestic pigs by an arthropod vector, or directly and indirectly between swine (Brown and Bevins 2018). Soft ticks of the genus *Ornithodoros* (Ixodida: Argasidae) can transmit ASFV to domestic, wild, or feral pigs through saliva while blood feeding. Direct transmission between swine occurs through contact between a susceptible animal and an infected animal's saliva, mucus, urine, or feces. Indirect transmission involves infective fomites such as undercooked pork products or contaminated animal feed; ASFV is a DNA virus that remains viable in the environment for weeks or months (McKercher et al. 1978, EFSA 2015), providing ample opportunity for indirect transmission.

We have identified three priority scenarios through which ASFV could be transmitted in the United States: (1) a sylvatic cycle between feral swine (*Sus scrofa*) and soft ticks, (2) spillover from the sylvatic cycle into domestic pig (*Sus scrofa domesticus*) populations through arthropod vector or contact with feral swine, and (3) direct transmission among feral or domestic swine. The presence of multiple transmission pathways adds complexity to disease eradication efforts as infected wild host and vector presence create persistent risk of spillover into domestic pig populations even after quarantine and depopulation of affected livestock.

Proactive strategies that reduce viral introduction and enhance the detection and response to outbreaks are key to protecting the health and economic stability of the United States and international trading partners. Understanding where the impacts of disease incursion may be most severe is crucial for protecting animal health. The risk of ASFV incursion in the United States varies spatially with the distribution of competent hosts and competent vectors (Brown and Bevins 2018). In anticipation of continued ASFV movement and emergence, we reviewed publicly available data on the distributions of domestic and feral swine in the United States along with soft tick (Argasidae) occurrence data based on prior published studies and museum collections. The objective of this study was to synthesize the distributions of vectors and hosts to predict the composite spatial risk of (1) ASFV establishment in a sylvatic cycle between feral swine and ticks, (2) spillover from the sylvatic cycle to domestic swine by an arthropod vector, and (3) direct transmission between feral or domestic swine. The resulting information provides valuable insights into predicted spatial risk of ASFV transmission through each of these three scenarios.

## Materials and Methods

### Data acquisition

County-level swine inventory in 2012 was gathered from the United States Department of Agriculture National Agricultural Statistics Service (USDA-NASS 2012) and includes all operations that would normally produce or sell at least $1,000 USD of swine during the census year. Domestic swine abundance was calculated directly for 866 of 3080 counties. To estimate missing values, we regressed the total county inventory for facilities of a certain size on the number of facilities and constrained the intercept to 0. We used the resulting regression slope as the average number of pigs per facility of that size. State-specific slopes were calculated whenever possible to accommodate between-state variation; otherwise, the national slope was used.

Feral swine occurrence records were gathered from the USDA-APHIS website (USDA-APHIS 2016). These data are collected through the National Feral Swine Mapping System by state wildlife agencies, USDA-APHIS Wildlife Services, and other interested parties. Records are updated continually with deletions of eliminated populations or addition of newly established populations (Corn and Jordan 2017). We used the final county-level map from the year 2016.

*Ornithodoros* soft tick collection records were (1) provided by United States National Tick Collection at Georgia Southern University, (2) gathered from the Global Biodiversity Information Facility (GBIF.org 2018), and (3) supplemented by published reports (Cooley et al. 1944, Kaiser 1966, Furman and Loomis 1984, Teglas et al. 2006). County-level records included 1332 collections between 1891 and 2018, and were entered into our database as present or not detected.

### Composite risk

Composite risk was calculated in three ways: (1) risk of sylvatic enzootic disease establishment, which requires the county-level co-occurrence of feral swine and soft ticks, (2) spillover risk from a sylvatic disease cycle to domestic pigs, which requires the county-level co-occurrence of feral swine, soft tick vectors, and domestic swine populations, and (3) risk of direct transmission among feral swine and domestic swine.

For those scenarios involving vectors, we consider only those *Ornithodoros* ticks that occur in the United States that have been experimentally shown to be capable of transmitting ASFV to swine—*Ornithodoros coriaceus*, *O. turicata*, and *O. puertoricensis* (Groocock et al. 1980, Hess et al. 1987, Endris et al. 1991, Endris and Hess 1992). Only members of the genus *Ornithodoros* have been implicated in ASFV transmission in any country, either in the field or in the laboratory. However, because a number of U.S. *Ornithodoros* tick species have not been tested for virus competence, we included separate choropleth maps for all reported U.S. species in this genus (Supplementary Figs. S1, S2, S3, S4) that might be integrated into this analysis if other soft tick species or genera were identified to serve as competent vectors.

To calculate the risk of sylvatic establishment, we combined feral swine and competent *Ornithodoros* tick occurrence to identify counties in which a sylvatic vector-borne disease cycle is possible. For those counties in which both ticks and feral swine co-occurred, we integrated the domestic swine inventory map to highlight domestic livestock populations at risk of spillover if establishment of a sylvatic disease cycle were to occur. Finally, we synthesized feral swine occurrence and domestic swine inventory maps to produce an index of direct swine-to-swine transmission risk. We used ArcGIS 10.5.1 (Redlands, CA) to visualize ASFV risk for all three scenarios across the continental United States.

## Results

### Host and vector occurrence summaries

The domestic swine inventory in 2012 included a county-level national mean of 19,578 ± 1517 standard error swine, a median of 248, and a range of 0 to 1,859,042. Domestic swine were reported in 2876 of 3080 counties; 204 counties reported none. Areas of particularly concentrated production were found in Midwest states and North Carolina.

In 2016, feral swine were documented in 1312 of 3080 counties and in 34 of 48 states in the continental United States. Feral swine populations range from sparse to dense depending on habitat quality and other environmental and historical factors; however, density estimates for feral swine do not exist for most of their range.

At least 21 species of soft ticks in the genus *Ornithodoros* were included in the U.S. National Tick Collection records: *O. aquilae* Cooley; *O. brasiliensis* Aragão; *O. brodyi* Matheson; *O. concanensis* Cooley and Kohls; *O. coriaceus* Koch; *O. denmarki* Kohls; *O. dugesi* Mazzoti; *O. dyeri* Cooley and Kohls; *O. hasei* Schulze; *O. hermsi* Wheeler, Herms and Meyer; *O. kelleyi* Cooley and Kohls; *O. nicollei* Mooser; *O. parkeri* Cooley; *O. peropteryx* Kohls and Clifford; *O. puertoricensis* Fox; *O. rossi* Kohls, Sonenshine and Clifford; *O. sparnus* Kohls and Clifford; *O. stageri* Cooley and Kohls; *O. talaje* Guèrin-Méneville; *O. turicata* Dugès; and *O. yumatensis* Cooley and Kohls. Most records occur in southern and western states. Of these species, three have been established in experimental studies as competent to transmit ASFV to swine: *O. coriaceus*, *O. puertoricensis*, and *O. turicata* (Groocock et al. 1980, Hess et al. 1987, Endris et al. 1991, Endris and Hess 1992). One other North American *Ornithodoros* species, *O. parkeri*, acquired infection, but was unable to transmit ASFV to swine (Hess et al. 1987). The other species remain uninvestigated.

### Composite risk assessment

#### Sylvatic cycle

**FIG. 1.**
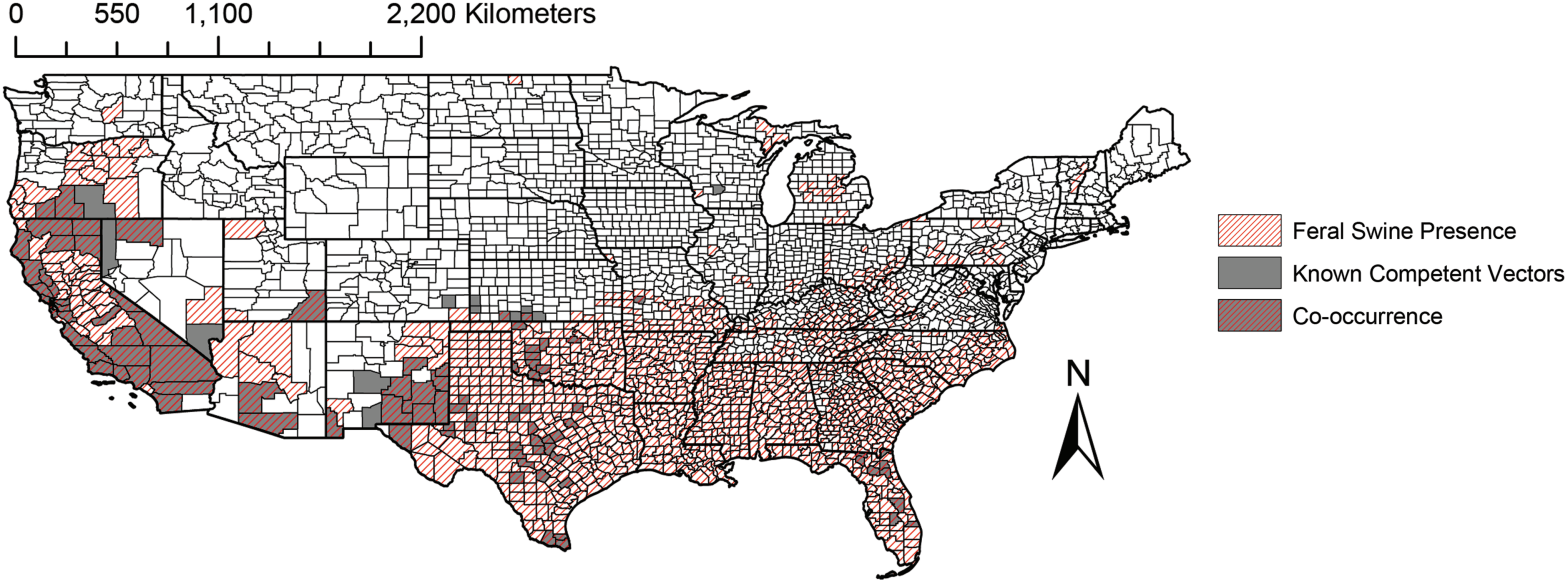
Choropleth map of ASFV sylvatic establishment risk based on co-occurrence of feral swine and competent tick species in the genus *Ornithodoros*. ASFV, African swine fever virus. Color images are available online.

Known competent ticks and feral swine co-occur in much of California, as well as in parts of Oregon, Nevada, Utah, Arizona, New Mexico, Texas, Oklahoma, Kansas, and Florida (Fig. 1). Consequently, the risk of sylvatic disease establishment after introduction appears highest in southwestern states and in Florida.

#### Spillover

**FIG. 2.**
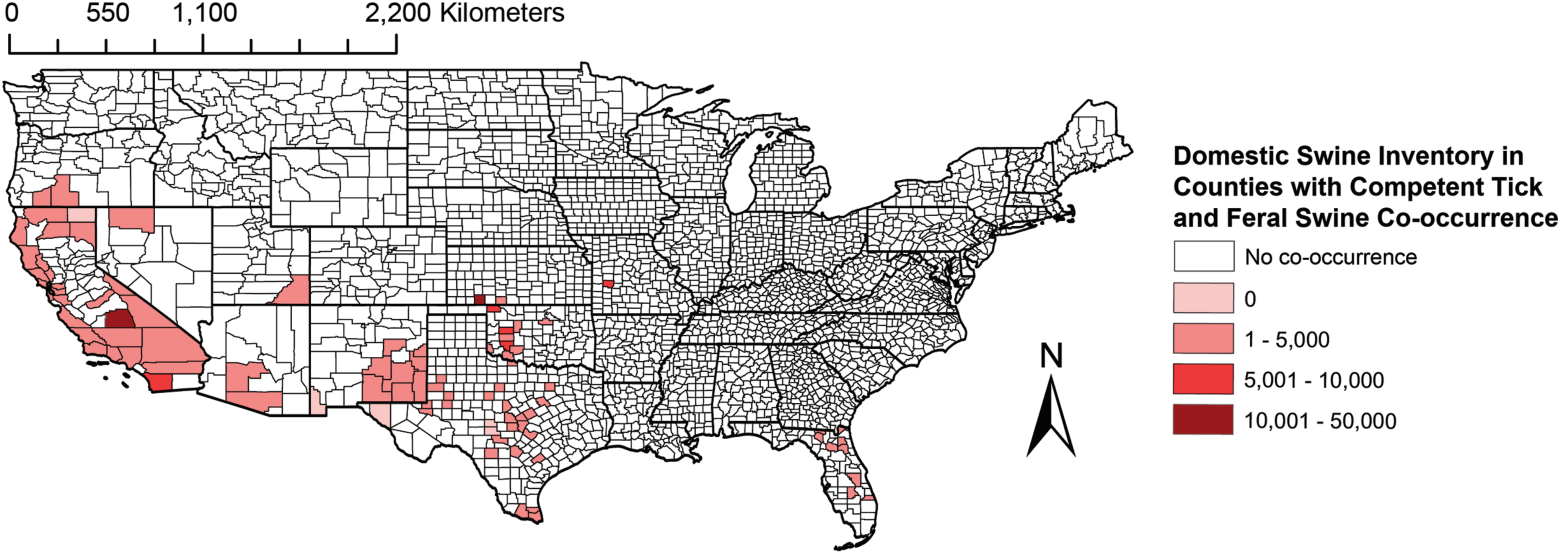
Choropleth map of ASFV spillover risk from a sylvatic disease cycle to domestic pigs based on co-occurrence of feral swine, competent *Ornithodoros* ticks, and density of domestic swine. *Unshaded* counties represent those without feral swine and competent tick co-occurrence. Color images are available online.

Within those counties with co-occurrence of known competent tick vectors and feral swine, domestic swine typically do not reach high inventory (Fig. 2). Areas of California, Oregon, Nevada, Arizona, New Mexico, Oklahoma, Texas, and Florida are potentially at risk of ASFV spillover due to the co-occurrence of *Ornithodoros* ticks, feral swine, and domestic swine. Relatively high domestic swine populations occur in California, Kansas, and Oklahoma counties where a sylvatic cycle is possible.

#### Direct transmission

**FIG. 3.**
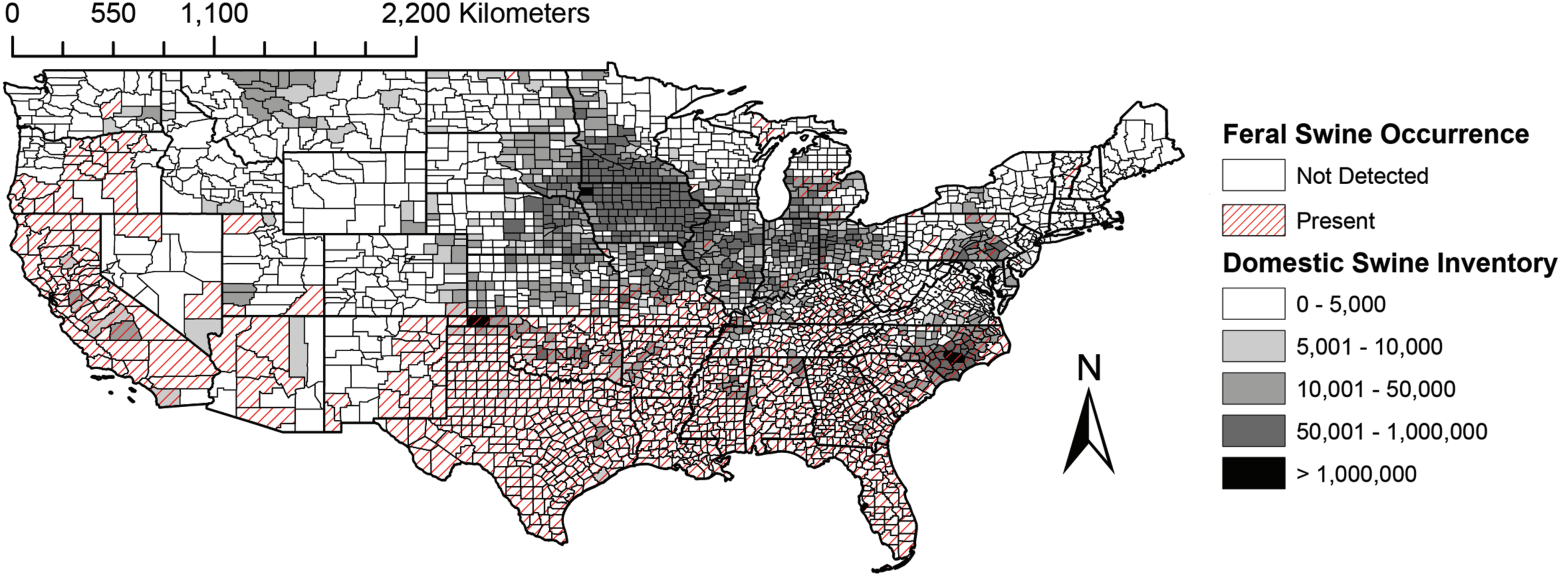
Choropleth map of ASFV direct transmission risk among feral and domestic swine based on occurrence of feral swine and domestic swine inventory. Color images are available online.

Much of North Carolina and parts of Oklahoma are of particularly of high concern for direct swine-to-swine virus transmission due to the presence of feral swine and high densities of domestic swine (Fig. 3).Virus transmission among feral and domestic swine may also be of particular concern in California, Texas, and other states in the southeastern United States.

## Discussion

This study determined the spatial risk for ASFV establishment and spillover in the United States and identified priority areas of future study to maximize preparedness for an introduction of ASFV. By compiling data on the distribution of susceptible animals and arthropod vectors, including feral swine, domestic swine, and competent *Ornithodoros* soft ticks, we identified much of the southern United States as potential hotspots for sylvatic disease establishment, spillover to domestic swine, and direct transmission between swine, should ASFV enter the country. Introduction pathways were not the focus of our analyses; however, California, Texas, Georgia, and Florida were the states of destination for 36% of U.S. goods imports by April of 2018 (11.4%, 11.5%, 4%, and 3.1%, respectively; USCB 2018). Furthermore, California, Texas, and Florida were among the states identified as at greatest risk for ASFV introduction by legal imports of infected swine or swine products (Herrera-Ibatá et al. 2017). These states were also highlighted as risk areas for the subsequent spread of ASFV after an introduction event in our analyses. Therefore, we recommend that proactive strategies for the reduction of ASFV risk such as inspection of imports and monitoring of susceptible populations be concentrated in these states.

*Ornithodoros* ticks are long lived, can survive for months or years without a meal (Sonenshine and Roe 2013), and can remain infected with ASFV for years (Kleiboeker and Scoles 2001). Furthermore, some *Ornithodoros* soft tick species can transmit the infection transovarially (Rennie et al. 2001) and sexually (Plowright et al. 1974); accordingly, ASFV could persist in tick populations even in the absence of infected hosts. Should ASFV become enzootic in U.S. soft ticks, continual long-term monitoring would be required to ensure that the disease will not emerge (or re-emerge) in swine.

Despite their potential importance, much remains unknown about the distribution, host preferences, and vector competence of U.S. soft tick species. *Ornithodoros* feed quickly, dropping off hosts within 20–70 min (Anderson and Magnarelli 2008). Accordingly, the common sampling technique of collecting ticks attached to captured vertebrate hosts is unlikely to contribute to the characterization of soft tick distribution or host associations. Furthermore, because *Ornithodoros* ticks do not quest for hosts, tick dragging, where a piece of cloth is mounted on a pole and dragged across suspected tick habitat, is also an ineffective soft tick sampling technique. Arthropod traps baited with carbon dioxide are effective in capturing soft ticks when placed near animal burrows (Caiado et al. 1990, Adeyeye and Butler 1991).

Our range maps are based on historical records, and the majority of collections likely represent areas of established occurrence; however, it is possible that ticks no longer occur in the areas reported. We recommend more extensive sampling of soft ticks across the United States paired with ecological niche modeling to explore soft tick distribution and likely host associations (Vial et al. 2018), as has recently been done for *O. turicata* and *O. hermsii* in the United States (Donaldson et al. 2016, Sage et al. 2017). We further recommend that more U.S. soft tick species be evaluated for ASFV vector competence.

Although overlapping spatial distributions are necessary for pathogen transmission between species, co-occurrence at the county level does not imply that species interactions will occur to facilitate transmission. For example, *Ornithodoros* soft ticks are nidicolous, generally remaining in host burrows or dens throughout development (Anderson and Magnarelli 2008). Except when farrowing, feral swine do not use dens but instead sleep in shallow depressions in leaves or loose earth (Graves 1984), so most contact between *Ornithodoros* ticks and feral swine is likely to be incidental and not habitual. However, the use of farrowing nests may place juveniles and adult females at higher risk of vector-borne ASFV transmission.

U.S. hog producers use a number of practices that minimize contact between livestock and feral swine or arthropod vectors, and are designed to prevent disease transmission between production groups. The vast majority of operations with 100 or more pigs hold animals in facilities with no outside access (>98%) and rigorous biosecurity measures, such as “all-in/all-out” (>96%), a management practice where facilities are emptied and disinfected before more animals enter (USDA 2015).

However, organic and small-scale operations remain at high risk for contact between livestock and infected hosts and vectors. To qualify for certified organic status, pigs must have access to the outdoors and direct sunlight year-round (USDA-AMS 2016). In 2011, there were 12,373 organic pigs reported in the United States, with the highest numbers in Iowa (4406 head), Wisconsin (1933 head), California (1810 head), and New Jersey (1000 head; USDA-ERS 2013). Competent tick vectors for ASFV are present in much of California, and feral swine are found in both California and Wisconsin, presenting a risk to organic domestic swine in these areas should ASFV outbreaks occur. Of swine operations with <100 pigs, 66.8%–76.8% allow animals access to the outdoors (USDA 2014). “Backyard” operations that produce <$1,000 USD each year were not included in the USDA-NASS data set. In addition, Vietnamese potbellied pigs (*Sus scrofa domesticus*) are popular pets in the United States (Van Metre and Angelos 1999), and may provide another category of competent reservoirs in the regions of risk.

Feral swine are a destructive invasive species in the United States, with a rapidly expanding range (Bevins et al. 2014, Corn and Jordan 2017). Expansion into regions with higher inventories of domestic swine may increase the risk of transmission of agents such as ASFV. Domestic pigs may contact feral swine on free range or organic operations and through sty fencing. Nationwide, nearly 8% of small-scale swine operations, and 3% of large-scale operations, reported feral swine sightings on site in the previous year (USDA 2014, 2015). These numbers are likely underestimates, as the primarily nocturnal activity of feral swine does not coincide with common human work periods (Graves 1984). In fact, some Texas feral swine appear to be attracted to domestic pigpens, especially those containing females (Wyckoff et al. 2009). Of domestic pig production sites that reported feral swine sightings, 16.4% and 11.7% of small- and large-scale operations, respectively, reported evidence that feral swine had gained access to domestic swine housing and feed-storage facilities.

Feral swine presence, but not density, appears to be an important risk indicator for ASFV introductions, as no correlation has been found between feral swine density and reported cases of ASFV in Europe (EFSA 2015, Bosch et al. 2017); however, density may drive feral swine dispersal, and could increase the likelihood of local disease establishment. Thus, studies on feral swine densities across the United States would augment our understanding of the potential spatial dynamics of ASFV and other high-consequence diseases should introduction occur.

In addition to feral swine, the common warthog (*Phacochoerus africanus*) and collared peccary (*Pecari tajacu*) are found in South Texas, where feral swine also occur. Warthogs can develop asymptomatic infection with ASFV after contact with infected soft ticks, although neither vertical nor horizontal transmission has been reported (Brown and Bevins 2018). Peccaries are generally considered resistant to ASFV (Brown and Bevins 2018), but data are lacking, and we recommend that the host competence for this species be evaluated more thoroughly.

This study combined data on the occurrence of feral swine, soft ticks, and the inventory of domestic swine in the United States to estimate risk of ASFV establishment in a sylvatic cycle, spillover from a sylvatic cycle into domestic swine, and direct transmission among feral or domestic swine. This synthesis highlights that regions with greatest risk of sylvatic transmission between feral swine and soft ticks and spillover to domestic swine from ticks include the southwest from California to Texas and regions of southern Florida (Figs. 1 and 2). Regions with risk of transmission from feral swine to domestic pigs include the southern half of the United States (Fig. 3). This study highlights several key knowledge gaps that limit the ability to predict the transmission of ASFV in the United States, principally *Ornithodoros* soft tick distribution and host association data.

## Supplementary Material

Supplemental data

Supplemental data

Supplemental data

Supplemental data

## Supplementary Material

Supplementary Figure1

Supplementary Figure2

Supplementary Figure3

Supplementary Figure4
