# Supplementary material for: Risk of African Swine Fever Virus Sylvatic Establishment and Spillover to Domestic Swine in the United States
Source: Vector Borne Zoonotic Dis. 2019 Jun 26;19(7):506–11. doi: 10.1089/vbz.2018.2386 (PMC6602112; doi:10.1089/vbz.2018.2386)
Supplement: Supplemental data [file Supp_Fig4.pdf]

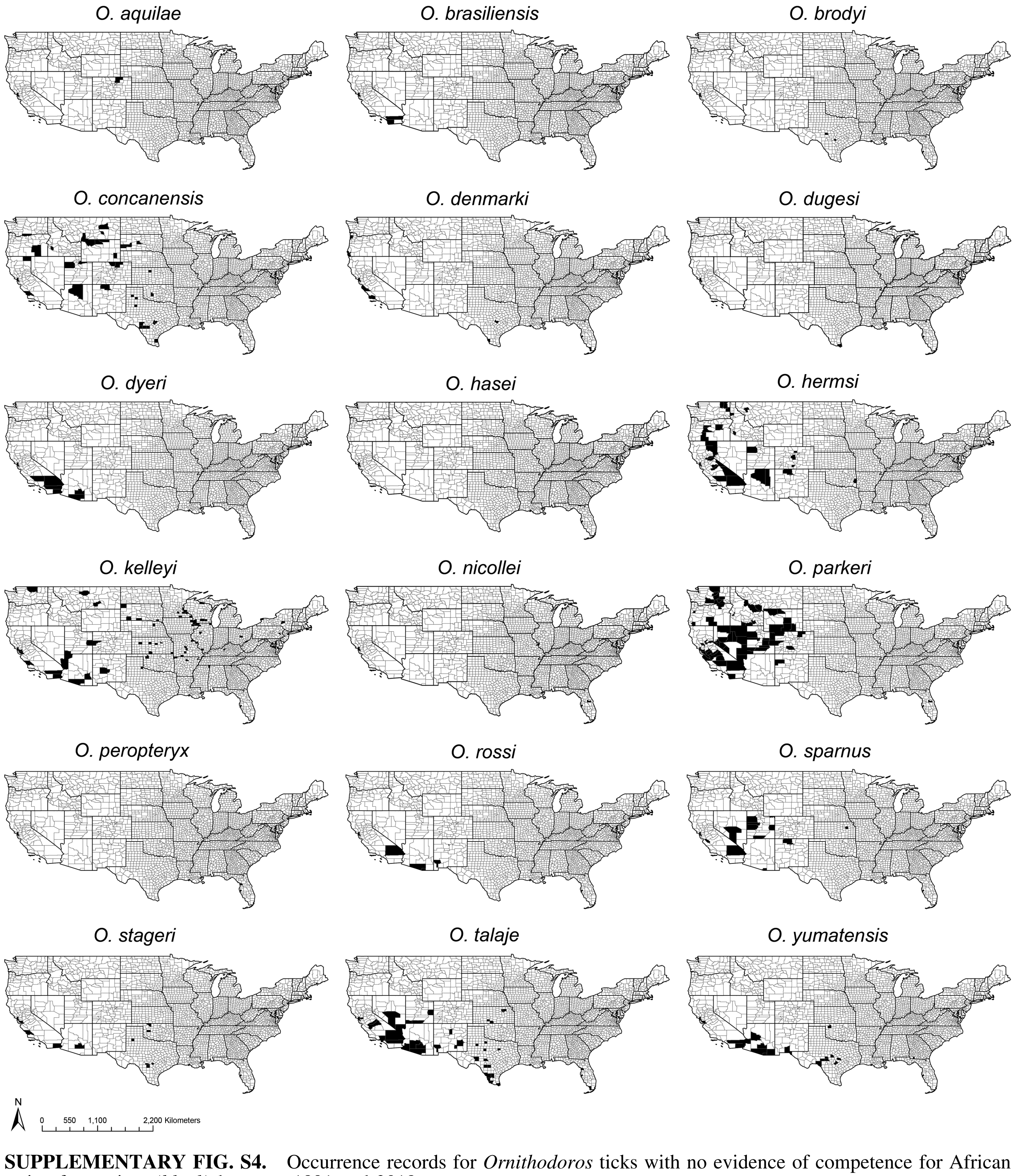

SUPPLEMENTARY FIG. S4. Occurrence records for *Ornithodoros* ticks with no evidence of competence for African swine fever virus (*black*) between 1891 and 2018.
